# Primary School Children's Health Behaviors, Attitudes, and Body Mass Index After a 10-Week Lifestyle Intervention With Follow-Up

**DOI:** 10.3389/fped.2018.00137

**Published:** 2018-05-09

**Authors:** Elise C. Brown, Duncan S. Buchan, Dorin Drignei, Frank B. Wyatt, Lon Kilgore, Jonathan Cavana, Julien S. Baker

**Affiliations:** ^1^Department of Public and Environmental Wellness, Oakland University, Rochester, MI, United States; ^2^Institute for Clinical Exercise & Health Science, University of the West of Scotland, Hamilton, United Kingdom; ^3^Mathematics-Statistics Department, Oakland University, Rochester, MI, United States; ^4^Department of Exercise Physiology & Athletic Training, Midwestern State University, Wichita Falls, TX, United States; ^5^Kilgore Academy, Azle, TX, United States; ^6^Health Improvement Department, National Health Service Lanarkshire, Carluke, United Kingdom

**Keywords:** school, children, obesity, attitudes, physical activity, body mass index, health, behavior

## Abstract

**Background:** Given the current global child obesity epidemic, testing the effectiveness of interventions in reducing obesity and its influencers is paramount. The purpose of this study was to determine immediate and long-term changes in body mass index and psychosocial variables following a 10-week lifestyle intervention.

**Methods:** Seven hundred and seventy participants (8.75 ± 0.98 years of age, 379 boys and 391 girls) took part in the study. Participants had height, weight, and psychosocial questionnaires assessed at pre- and post-control, pre- and post-intervention, and 6-months post-intervention. Participants completed a weekly 10-week intervention consisting of healthy eating and physical activity education, physical activity, parental involvement, and behavior change techniques. Regression models were fit with correlated errors where the correlation occurred only between time points, not between subjects, and the nesting effects of school and area deprivation were controlled.

**Results:** Regression models revealed a significant decrease in body mass index from pre- to post-intervention of 0.8512 kg/m^2^ (*P* = 0.0182). No Changes in body mass index occurred from post-intervention to 6-month follow-up (*P* = 0.5446). The psychosocial variables did not significantly change.

**Conclusions:** This lifestyle intervention may be an effective means for improving body mass index in primary school children in the short-term if the duration of the intervention is increased, but these changes may not be sustained without on-going support.

## Introduction

It is well-established that healthy eating (HE) and physical activity (PA) are associated with the prevention and treatment of child obesity (1, 2). While food preferences are linked to HE, other factors are associated with HE and PA in youth including environment, socioeconomic status, family involvement, and attitudes (3, 4). Theoretical models that many lifestyle interventions are based on suggest that improving attitudes while supporting individuals at multiple levels may enhance HE and PA behaviors resulting in improved weight status (5). However, there has been a lack of long-term follow-up at the conclusion of many of these interventions investigating any sustained effects on obesity as well as health attitudes and behaviors (1, 2).

Schools are considered ideal settings for improving child health behaviors because of the amount of time that children spend there, infrastructure, and the role that schools play in community education and health (6). Although school-based interventions have had a moderate impact on improving fruit intake in children, increasing vegetable intake may be challenging (7, 8). Nonetheless, increasing fruit and vegetable intake may aid in obesity prevention as it has been demonstrated that higher consumption can lead to weight loss (9) and provide other health benefits (10). Evidence from school-based lifestyle interventions addressing obesity indicates that interventions with HE and PA components alongside parental involvement are consistent with weight reduction in children with body mass index (BMI) often used as the outcome measure (11).

It is important for school-based interventions to be theoretically informed in order to identify variables influencing behaviors (12) and provide information on designing interventions to manipulate these variables for behavior change (13). Improving health attitudes in children are important because attitudes may predict behavior as demonstrated in school-based interventions (14, 15). This is consistent with the Theory of Reasoned Action/Theory of Planned Behavior (TRA/TPB) which seeks to explain volitional behaviors partly through attitudes (16). School-based interventions have achieved improved health attitudes and behaviors (14, 17). It is important to test this theory alongside other health behavior models in determining the immediate and sustained impact of school-based lifestyle interventions (18).

A school-based lifestyle intervention, Fit for School (FFS), sought to improve the weight status of children by positively influencing HE and PA attitudes and behaviors. Thus, the aim of this evaluation was to determine the effectiveness of FFS on immediate and long-term changes in BMI and psychosocial variables.

## Materials and methods

### Participants

**Figure 1:**
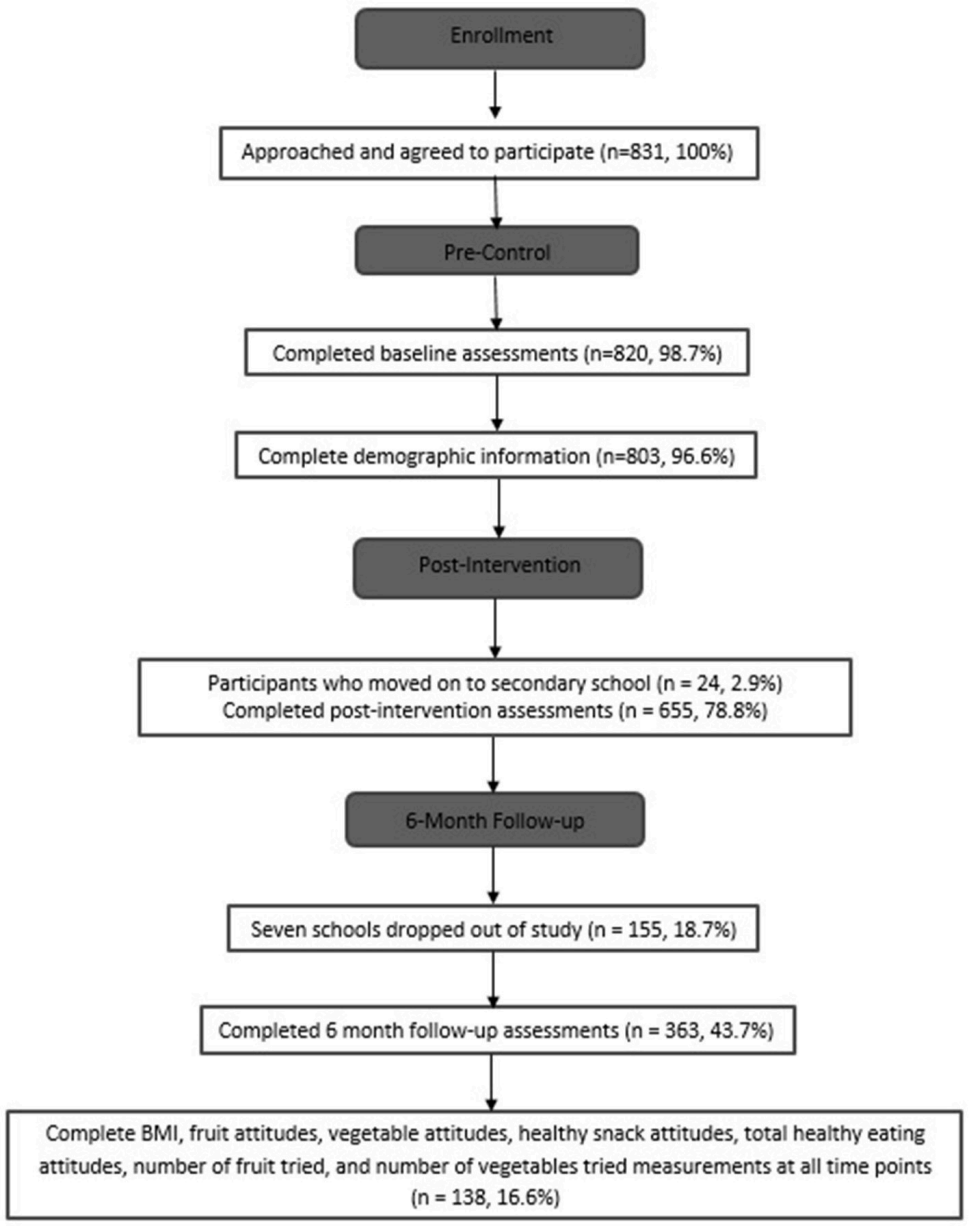
Flow of participants through the study.

Figure 1 shows the flow of participants through the study. A total of 831 participants agreed to participate in the study and ranged in age from 6 to 12 years in Lanarkshire, Scotland. This study was part of a Child Healthy Weight program evaluation in Scotland commissioned by National Health Service Lanarkshire. FFS was developed by this health organization and has a wide reach which is now being evaluated. Many interventions are currently implemented by schools without any evaluations of their benefits, and this is what this study and findings are hoping to address. Schools were recruited across all of Lanarkshire and selected based on interest, availability, and class sizes. It is possible that because these schools were partly chosen on expressed interest, the selected schools may have been more likely to offer a greater degree of support for these types of lifestyle programs compared to schools that did not express interest. This greater level of support from school administrators may have impacted how the participants responded to the program (19). The schools were from a range of deprivation areas as measured by the Scottish Index of Multiple Deprivation (SIMD) (20). Ethical approval was obtained from National Health Service Lanarkshire. Information and consent forms were sent home with the children prior to the study, and parental consent and participant assent were received from all participants prior to the study.

### Study design

A repeated measures design was utilized such that each participant served as a control from January 2013–March 2013, then participated in the intervention from April 2013–June 2013, and then completed follow-up measurements at a 6-month time point post-intervention. Given this was a Scottish Government intervention with certain logistical constraints in terms of delivery, a randomized controlled trial was not possible. Although randomized controlled trials characterized by a 1:1 ratio of participants randomized to a treatment and a control group offer the benefits of reducing confounding factors, selection bias, and interpretation bias, a repeated measures design decreases the variability between participants resulting in enhanced statistical power with a requirement of fewer participants (21). A limitation to the repeated measures design, however, is a lack of control of order effects. A multi-agency interdisciplinary partnership approach was used to recruit schools for participation and to provide participants with ongoing support after the intervention (22) including providing participants with information on extracurricular PA programs hosted by Leisure Trusts (23).

### Intervention

FFS was a 10-week intervention with weekly sessions lasting 90 min and included HE and PA education, PA activity sessions, parental involvement, and behavior change components. The primary intervention aim was to increase the proportion of children classified as healthy weight. FFS was delivered to the entire class in order to help those outside of a healthy weight range achieve a healthy weight and also to support those currently with a healthy weight to maintain their weight status. The intervention was delivered by healthy lifestyle coaches trained in health behavior change theory with a focus on supporting student engagement, motivational interviewing techniques, FFS ethos, content and delivery, height and weight measurement, and information governance. The 45-min education lesson aligned with the Scottish Curriculum for Excellence (23). The 45-min PA session consisted of fun-focused games, exercises, and sports, such as tag and soccer, that were designed to keep the children moving the majority of the time. Weeks 1 and 10 of FFS included an introduction and review of the intervention, respectively, and assessments were also conducted during these weeks. The program was divided into 7 age-specific units. Sub-topics varied with each age group to ensure age-appropriate material. For example, HE topics for younger children in Primary 1 (aged 4–6 years) included an understanding that people need more of some foods and less of others, while topics for older children in Primary 7 (10–12 years) included how different foods and drinks have energy and different nutrients in varying amounts. Individual units were made up of 6 thematic modules including PA, HE (diet and health, food and drink choices, food and drink sources, consumer awareness, food and drink experience), physical education (PE), healthy lifestyle topics, home-link, and class projects, and were delivered over 8 weeks. See Brown et al. (24) for more information on study protocol related to the FFS program.

The combination of three different behavior change theories were used in FFS including TRA/TPB (16), Transtheoretical Model of Behavior Change (TTM) (25), and Social Ecological Model (SEM) formatted in a health education framework (19). Children were supported while moving through the TTM stages of change at multiple levels including school, home, and after-school leisure center PA programs which paralleled assumptions of TRA/TPB which allow children to go through a conscious process deciding the benefits of changing behaviors and then designing individual plans about making specific changes through goal setting. The multiple level support reflects the premise of the SEM which postulates health behaviors are the result of a dynamic interplay among the individual, their relationships, the community, and societal factors (26). In efforts to promote student interaction, coaches employed a motivational interviewing technique which has been demonstrated to be an effective approach to improving weight status in children (27, 28), Through motivational interviewing, the coaches aimed to induce self-reflection and elicit positive motivation for change statements which may lead to self-liberation, a strong commitment for change and an integral process of change construct of the TTM (25). Parental involvement included passive strategies such as signing consent forms, FFS information sheets, and weekly homework assignments that participants completed with parents. An example of one of the homework assignments to be completed with parents was deciding on health behavior change goals. Although more active parental involvement strategies such as cooking workshops may be more effective for improving dietary intake than passive strategies, the approaches used in the current study may be more sustainable in the long-term for a school-based setting (29).

### Procedures

Participants completed height, weight, and questionnaire assessments at the following time points: pre-control, post-control, pre-intervention, post-intervention, and 6-month follow-up. Participants completed the questionnaires independently although the lifestyle coaches were available to clarify any queries participants had.

### Measures

#### Height and weight

Each coach was trained to measure height (cm) and weight (kg) based on the Child Measurement Program Operational Guidance (30). Body weight was measured in kilograms (kg) using SecaTM 899 digital scales. Participants were weighed in light clothing standing with both feet in the center of the scale without shoes. The values were recorded by the coach and rounded to the nearest 0.1 kg. The Seca Leicester Height Measure stadiometer was used to assess height rounded to the nearest 0.1 cm and participants were not wearing shoes.

#### Pupil questionnaire

The Pupil Questionnaire (9 constructs with multiple items each), previously used in a large-scale UK government evaluation of the School Fruit and Vegetable Pilot Scheme, was used to assess HE attitudes and behaviors (31). Validity and reliability were assessed using data from the control period of the present study, and the Pupil Questionnaire demonstrated sufficient test-retest reliability [intraclass correlation coefficients (ICC): 0.67–0.88] and nomological validity (*P* = 0.03) in youth aged 6–12 years for the following variables: number of fruits tried (ICC = 0.67; lower and upper 95% confidence intervals: 0.61–0.72), number of vegetables tried (0.71; 0.66–0.75), fruit attitude (0.87; 0.84–0.89), vegetable attitude (0.88; 0.86–0.90), healthy snack attitude (0.74; 0.70–0.78), total HE attitude (0.84; 0.82–0.87), and portion of fruit knowledge (0.67; 0.57–0.75). The psychosocial variables investigated in the present study included fruit attitudes, vegetable attitudes, healthy snack attitudes, total HE attitudes, number of fruit tried, and number of vegetables tried.

### Data analysis

#### Data cleansing

Quality control measures were taken to minimize unfeasible data as the result of measurement error. Exclusion criteria for analysis was based on a combination of biological and statistical likelihood similar to Berkey and colleagues' approach (32). Any child whose height changed <-2.7 cm from pre to post was excluded from the analyses (33). In accordance with Berkey and colleagues' approach, (32) participants whose change in BMI exceeded >3 *SD* beyond age and sex specific means from pre to post, post to 6 months, or post to 24 months were excluded from analyses. Children with heights declining >2.54 cm at 6 months and declining > 0 cm at 24 months were excluded from analyses. Additionally, children whose height increased >3 *SD* beyond age and sex specific means from post to 6 months were excluded from analyses. A total of 11 participants were removed from analyses as a result of this process.

### Statistical analyses

In this analysis the larger data sets with missing values were considered in an effort to increase the sample size for analysis. Regression models were fit with correlated errors to these data sets, where the correlation occurred only between time points, not between subjects. Separate models were considered for “Control” data (pre/post), “Intervention” data (pre/post), “Intervention” data (post/6 mo), and “Intervention” data with three time points (pre/post/6 mo). The magnitude and sign of the estimated regression coefficient for the “Time” variable would indicate whether there is a decrease or increase in the response variable across time, while the *p*-value indicates if this decrease/increase is statistically significant. Besides “Time,” the other regression variables are: Sex, Age, SIMD, School (SIMD), and their interactions with “Time.” Here “School” was nested in “SIMD.” The response variables were (separately): BMI, number of fruit tried, number of vegetables tried, fruit attitude, vegetable attitude, healthy snack attitude, and HE attitude. For each response variable, only the children who had at least one non-missing value in both “Control” and “Intervention” were retained. This strategy leads to a common sample of children for “Control” and “Intervention” in each response variable. Proc “mixed” in SAS with separate autoregressive time-correlation for each of the five SIMDs was used to fit the regression models. In order to determine if there were differences across all variables of interest between those participants who completed the intervention at all-time points and those who did not, a one-sided *t*-test was computed comparing the two groups at the pre-intervention time point. Extreme outliers were removed using boxplots (34). Based on the data in Brown et al. (35) using two independent samples of sizes 10 and 9 with BMI control vs. intervention mean difference of 1.24 (common standard deviation 3.4), and setting alpha to 0.05 and power to 80%, it is estimated that at least 120 subjects per group are needed for a *t*-test. The sample sizes used in this paper meet this criterion (35).

## Results

### Preliminary findings

Table 1 presents the demographic and baseline characteristics of participants included in analyses with valid BMI data. A total of 831 participants agreed to participate in the study, and 820 completed measurements at pre-control. Throughout the study, a small proportion of these participants left primary school to attend high school. There were 619 (74.5%) participants at pre- and post-control, 535 (64.3%) participants at pre- and post-control and pre- and post-intervention, and 378 (45.5%) participants at all-time points who attempted parts of the questionnaires. Out of the 378 participants, 246 participants had BMI measurements at all-time points. Eleven participants were removed as a result of data cleansing. After data cleansing procedures, 145 participants had valid BMI and questionnaire measurements at all-time points. After extreme outliers were removed, there were 138 participants (8.67 ± 0.51 years of age) who had valid BMI and questionnaire measurements at all-time points. There were no significant difference between participants who completed assessments all time points and those who did not across all of the variables of interest.

**Table 1:** Demographic and baseline characteristics of participants with valid body mass index data.

	**% (*n*)**
**AGE (YEARS)**	
6.00–7.99	10.4 (42)
8.00–8.99	65.8 (266)
9.00–9.99	21.5 (87)
10.00–10.99	2.2 (9)
**SEX**	
Boys	50 (202)
Girls	50 (202)
SIMD^*^	
1	29.5 (119)
2	10.6 (43)
3	41.3 (167)
4	6.9 (28)
5	11.6 (47)
WEIGHT STATUS^**^	
Overweight or obese	23.5 (95)
Normal weight or underweight	76.5 (309)

* *Scottish Index of Multiple Deprivation (SIMD) quintiles = 1 represents the most and 5 represents the least deprived areas*.

** *Weight status was based on the International Obesity Task Force cut-offs*.

Out of the 33 classes that enrolled in the study, 16 classes did not complete BMI measurements at all-time points and the schools were from a range of deprivation areas (SIMDs 1, 2, 3, and 5 with 1 representing the most deprived and 5 representing the least deprived areas).

### Main analysis

The results are presented in Table 2. The “BMI” row indicates that 404 children were analyzed for this response variable. There was no change in “Control” BMI from “pre” to “post” (*P*-value = 0.8507), and there was a significant decrease in “Intervention” BMI from “pre” to “post” of 0.8512 kg/m^2^ (*P* = 0.0182). There was no change in “Intervention” BMI from “post” to “6-months” (*P* = 0.5446), nor was there a change from “pre” intervention to 6-month (*P* = 0.0664).

**Table 2:** Estimated body mass index and questionnaire variables regression coefficients for “Time” and *P*-values.

**Response variable**	**Control (pre/post) time coefficient:**	**Intervention (pre/post) time coefficient:**	**Intervention (pre/6 mo) time coefficient:**	**Follow-up (post/6 mo) time coefficient:**	**Sample size**
	**Estimate (*****P*****-value)**	**Estimate (*****P*****-value)**	**Estimate (*****P*****-value)**	**Estimate (*****P*****-value)**	
Body mass index	0.0799(0.8507)	−0.8512(0.0182^*^)	−0.7871(0.0664)	−0.5212(0.5446)	404
Fruit tried	−0.7609(0.3445)	−0.2680(0.6848)	−0.1459(0.8208)	0.4433(0.7011)	769
Vegetable tried	−1.6220(0.0976)	1.0843(0.1888)	1.0486(0.1659)	1.9145(0.1214)	769
Fruit attitude	−0.0578(0.2568)	−0.0387(0.4146)	−0.0108(0.8150)	−0.0234(0.7935)	768
Vegetable attitude	−0.0740(0.1802)	0.0536(0.3204)	0.0218(0.6772)	−0.0127(0.8941)	770
Healthy snack attitude	−0.2195(0.0477^*^)	−0.0656(0.5209)	−0.0216(0.7983)	0.0407(0.8078)	739
Healthy eating attitude	−0.2905(0.0752)	−0.0222(0.8790)	−0.0285(0.8406)	0.0417(0.8813)	720

* *Indicates significance at the P < 0.05 level*.

For the questionnaire variables, a significant decrease in “Control” Healthy Snack Attitudes occurred from “pre” to “post” (*P* = 0.0477). None of the “Intervention” nor “Follow-up” Time coefficients for the remaining response variables were statistically significant. While no changes in “-tried” and “-attitude” response variables were individually statistically significant, collectively (and along with other factors) may have contributed to a statistically significant decrease in the “Intervention” pre/post BMI as suggested by the regression coefficients in Table 2.

## Discussion

### Main findings of this study

The purpose of this evaluation was to determine the effectiveness of FFS on the long-term changes in BMI and psychosocial variables following a 10-week lifestyle intervention. A significant decrease in BMI occurred from pre- to post-intervention and there was no change from post-intervention to 6-month follow-up. For the psychosocial variables, although there were no significant effects of the intervention, during the control period, there was a significant decrease in healthy snack attitudes suggesting that attitudes may worsen without appropriate lifestyle interventions in place.

The improvement in BMI during the intervention period was similar to another short-term school-based intervention (36). A 12-week health and nutrition education and exercise intervention to reduce the risk of type II diabetes resulted in a decrease in BMI in the intervention group compared to control. Similar to the current study, this was a specialist-led rather than a teacher-led intervention. It may be that having individuals other than the teacher delivering the intervention improved the implementation due to added resources and the novelty effect which may have increased interest (37). Although a systematic review by Brown and Summerbell (1) determined that intervention duration did not have an impact on effectiveness, other reviews have suggested that a minimum of 1 year is recommended to observe meaningful changes in BMI (2, 11). Given the short duration of the current intervention, it may be reasonable to expect that increasing the duration of FFS may allow more time for healthy eating attitudes and behaviors to improve which may have a more substantial impact on BMI.

No changes occurred in the number of vegetables nor fruit tried. It may not be sufficient to rely strictly on HE curriculum over such a short time period in order to expect changes in HE behaviors. For example, in the U.S. the Wellness, Academics, and You, a 12-month multidisciplinary obesity reduction intervention, resulted in significant increases in fruit and vegetable consumption (38). The Wellness, Academics, and You program included intensive parental involvement such as interviewing parents about family health history, a cross-curricular approach with integration with PE classes, and suggestions for changing school policy including improving food services. It is possible that if the duration of the current intervention was increased to at least 12 months and more intensive parental involvement strategies were implemented, an increase in number of vegetables and fruit tried may have occurred.

Attitudes did not change over the course of the intervention nor after follow-up. It may be that the intensity, including parental involvement strategies, and duration of the intervention may not have been sufficient to significantly impact vegetable attitudes, and sustained support may be necessary to maintain improvements long-term. In a 12-week gardening and HE education intervention that included multiple active learning strategies implemented several days each week, Duncan and colleagues noted an improvement in attitudes alongside an increase in fruit and vegetable intake (14). It may be that more intensive efforts including more frequent active learning strategies are necessary to improve HE attitudes.

The lack of sustained effects on vegetable attitudes may be due to the fluid nature of attitudes as they tend to vary within an individual over time and given the context (39). The SEM proposes that in order for health behaviors to be sustained stable changes need to occur at multiple levels (19). Long-term follow-up after the cessation of interventions is critical in determining if sustained changes in cognition and behaviors have occurred (40), but reviews of school-based interventions have reported very few studies including long-term follow-up assessments (1, 2). The follow-up findings of the present study highlight the need for continuous multicomponent interventions with support at multiple levels in order to maintain positive health attitudes and behaviors.

A limitation of the present study is the lack of completeness of data and the attrition rate. The attrition rate was in line with Reinehr and colleagues' 2-year follow-up study of clinical lifestyle interventions who were only able to obtain complete data in 8% of their participants (40). A second limitation was the lack of PA assessment. Inclusion of this measure would give an indication of the effects that the intervention may have had on PA.

A strength of the study is the within-subjects design in which the same participants served as both the control and intervention participants. This design decreased the variability between participants which, in turn, increased the statistical power (21). Because of the enhanced statistical power, fewer participants were necessary to determine an effect. Another strength was the 6-month follow-up. Conducting long-term follow-up measures is important for determining sustained intervention effects, identifying intervention strategies that may need altering, determining what causes a change in behavior, exploring intervention impact on a greater scale, and recognizing critical times for intervention delivery pertaining to long-term health (41).

## Conclusions

FFS was effective in improving BMI in primary school children in the short-term. If FFS is extended in duration, the program may be an effective means for improving HE attitudes and behaviors in primary school children in the short-term, but these changes may not be sustained without on-going support. Incentives should be put in place in order to aid in the prevention of school study attrition after the school has already received the intervention. Maintaining support post-intervention is necessary for sustained behavior change.

## Datasets are available on request

The raw data supporting the conclusions of this manuscript will be made available by the authors, without undue reservation, to any qualified researcher.

## Ethics statement

Ethical approval was obtained from NHS Lanarkshire. Parental consent and participant assent were received from all participants prior to the study.

## Author contributions

EB: Supervised all data collection, assisted with study design, and was a major contributor in writing the manuscript; DB and JB: Helped supervise data collection and were contributors to writing the manuscript; LK: Planned the study design and data collection procedures; FW and DD: Analyzed and interpreted the data regarding the changes in body mass index, attitudes, and behaviors. All authors read and approved the final manuscript.

### Conflict of interest statement

The authors declare that the research was conducted in the absence of any commercial or financial relationships that could be construed as a potential conflict of interest.

## Abbreviations

BMI: Body Mass Index
FFS: Fit for School
HE: Healthy Eating
ICC: Intraclass Correlation Coefficient
PA: Physical Activity
PE: Physical Education
SIMD: Scottish Index of Multiple Deprivation
TTM: Transtheoretical Model of Behavior Change
SEM: Social Ecological Model
TRA/TPB: Theory of Reasoned Action/Theory of Planned Behavior
